# Adaptive frequency-domain CFAR for robust spectrum sensing under jamming and administrator-controlled counter-access

**DOI:** 10.1038/s41598-026-48876-7

**Published:** 2026-04-27

**Authors:** Mohamed Salah Shams, Ahmed A. Abouelfadl, Ahmed Mansour, Mohamed Samir Abdel Latif Soliman

**Affiliations:** 1 Electrical Engineering Branch, Military Technical College, Cairo, Egypt; 2 Electronic Warfare Department, Military Technical College, Cairo, Egypt

**Keywords:** Cognitive radio, Spectrum sensing, Energy detection, CFAR, Jamming, Secure access, Engineering, Mathematics and computing

## Abstract

Cognitive radio networks (CRN) enable secondary users (SUs) to opportunistically access underutilized licensed spectrum while protecting primary users (PUs) from interference. However, robust spectrum sensing under heterogeneous interference and noise uncertainty remains challenging. Conventional energy detection, matched filtering, and cyclostationary approaches either require extensive prior knowledge or degrade under interference, leading to excessive false alarms and service denial. We propose an adaptive frequency-domain constant false alarm rate (CFAR) spectrum sensing approach that dynamically sets frequency-specific thresholds based on neighboring spectral samples. This enhancement requires minimal prior knowledge. To address potential security risks from untrusted SUs exploiting this resilience, we introduce a centralized counter-access mechanism: an administrator-controlled jammer that defeats CFAR-equipped adversaries, preventing unauthorized access during emergencies. It safeguards primary communications by creating strategic frequency gaps that avoid known jammed PU channels. Using APCO Project 25 waveforms for PUs, Monte Carlo simulations evaluate cell-averaging (CA), greatest-of (GO), smallest-of (SO), order-statistics (OS), and censored CFAR variants across different channel models combined with multiple jamming scenarios. Results show OS and censored CFAR variants best stabilize false alarm rates and enhance detection in heterogeneous conditions, outperforming CA and GO/SO schemes. The comb-sweep jammer effectively denies service to CFAR-equipped SUs without impacting PUs, though at the expense of reduced spectrum availability. Sensitivity analysis confirms robustness across varied CFAR window configurations, precluding parameter-based evasion by unauthorized SUs. This work frames spectrum sensing and jamming in CRNs as a two-sided interaction between electronic protection for secondary users and administrative electronic attack for controlled denial.

## Introduction

The proliferation of wireless devices and services has led to a critical issue of spectrum scarcity. Despite this, a significant portion of the licensed radio spectrum remains underutilized for large periods of time. CR networks were developed as a promising solution to this problem, enabling unlicensed SUs to opportunistically access licensed spectrum bands when they are not in use by PUs. This dynamic spectrum access paradigm hinges on a fundamental capability: spectrum sensing. SUs must reliably detect weak PU signals and vacate the channel immediately upon their return to prevent harmful interference, which could range from degraded quality of service to critical failures in public safety or military communications.

Before delving into the details of the proposed CFAR approach, it is important to conceptualize it within the broader landscape of spectrum sensing techniques. Energy detection, the most widely adopted method in CR systems, is a blind technique requiring no prior knowledge of PU waveforms^1^. Although simple and widely used, its reliance on fixed thresholds makes it particularly sensitive to noise and interference. For this reason, it is often treated as the baseline against which more advanced techniques are compared, and we return to it later when motivating CFAR-based enhancements. Other sensing approaches present different trade-offs^2^. The matched filter detector, for instance, offers optimum detection performance by maximizing the signal-to-noise ratio. It achieves this by correlating the received signal with a locally generated replica of the known PU’s waveform, which is the optimal linear filter for maximizing the output SNR. This achieves the best linear performance but is only practical when the PU’s waveform is perfectly known, a condition rarely satisfied in opportunistic access scenarios^3,4^.

Conversely, cyclostationary feature detection exploits the unique periodic statistical properties of modulated signals to differentiate them from noise^5^. This method is highly robust to noise uncertainty, effectively overcoming the ”SNR wall” that plagues energy detectors, but it comes at the cost of high computational complexity and the need for large sample sizes^1^. Similarly, eigenvalue-based detection utilizes the statistical properties of the received signal’s covariance matrix to detect the presence of a structured signal without relying on an absolute energy reference^6^. It is a blind technique that is insensitive to noise power uncertainty^7^, yet it also presents a significant computational burden due to the need for covariance matrix computation and eigenvalue decomposition^8^.

As noted earlier, conventional energy detection serves as the baseline method for spectrum sensing but suffers from fundamental limitations due to its reliance on a fixed detection threshold. In practice, the noise power experienced by a receiver often fluctuates because of temperature variations, hardware imperfections, or environmental changes^9^. When this occurs, the predetermined threshold no longer reflects the true operating conditions, leading to a trade-off between excessive false alarms that deny SUs legitimate access and missed detections that risk harmful interference with PUs. This inherent unreliability motivates the adoption of adaptive thresholding strategies. A particularly effective family of such methods is constant false alarm rate (CFAR) detection, which has been widely employed in radar systems for decades. In that context, CFAR dynamically adjusts the threshold by estimating the local noise or clutter level, thereby maintaining a stable false alarm rate across heterogeneous environments^10,11^. Traditional radar CFAR operates in the time domain by analyzing temporal sequences of range bins to characterize the interference background^12,13^.

Inspired by this proven effectiveness, we adapt CFAR principles to the frequency domain for spectrum sensing, enabling secondary users to robustly detect spectral opportunities even under uncertain and varying interference conditions. In particular, by dynamically adjusting the detection threshold based on real-time interference estimation, CFAR detectors maintain a consistent false alarm rate regardless of varying noise or jamming conditions. This transforms energy detection into a robust method suitable for modern CR applications while keeping its simple structure. There are two main families of CFAR algorithms: (a) mean-level estimators, such as Cell-Averaging CFAR (CA-CFAR) and variants that take the greatest or smallest of leading and lagging reference windows (GO-CFAR, SO-CFAR), which use averaging but differ in outlier handling^14^, and (b) order-statistic estimators, such as OS-CFAR (rank-based) and Censored CFAR (which discards the highest outlier samples), offering robustness against heterogeneous interference^15,16^. We leverage a range of these CFAR variants to ensure robust sensing under various jamming scenarios. However, spectrum sensing in CRNs is not merely a technical challenge of coping with noise and uncertainty; it is also a contested arena where malicious users may exploit these very mechanisms. Thus, while CFAR-based detection strengthens cooperative sensing, the story does not end here–we must now turn to the defensive side of the game, where the CRN itself must withstand adversarial attacks.

In this work, we propose the use of CFAR-based detectors to equip secondary users with spectrum sensing schemes that are inherently more adaptive to heterogeneous noise and interference conditions than conventional fixed-threshold methods. While this design strengthens the resilience of legitimate SUs, it also introduces a new security consideration: a malicious or untrusted SU could exploit the same robustness to maintain unauthorized access even when it should be denied. This paradox illustrates the two-sided game that characterizes cognitive radio networks–every enhancement in electronic protection simultaneously reshapes the landscape of electronic attack. Real-world conflict scenarios underscore this dual-use dilemma. For example, Ukrainian forces have leveraged CRs that sustain communications under heavy Russian jamming, with AI-enabled drones autonomously sensing interference and switching channels to maintain connectivity despite hostile conditions^17^. Such resilience is vital for legitimate use, but the very same techniques could be harnessed by adversaries. Similarly, a malicious UAV might transmit covertly within a PU’s band, making its signal indistinguishable from legitimate PU activity and thus evading detection^18^. These examples highlight that CFAR-enhanced robustness, while beneficial, cannot be considered in isolation–it must be balanced with mechanisms that allow administrators to retain control against empowered but potentially untrusted SUs.

These scenarios motivate our second contribution: an administrator-controlled jamming strategy that can defeat even the proposed CFAR-equipped SUs. Recent work has demonstrated that *intelligent*, algorithm-aware jamming substantially outperforms brute-force interference in dynamic spectrum environments. Zhang and Wu^19^model cooperative jamming against deep-reinforcement-learning-based frequency hopping as a Stackelberg game, showing that exploiting knowledge of the adversary’s decision logic yields significantly higher disruption rates than conventional power-flooding. Wang et al^20^. design and hardware-verify an intelligent jammer that tracks dynamic spectrum access patterns and intercepts opportunistic transmissions adaptively. Building on this principle of algorithm-awareness, we design a smart comb-sweep jammer that exploits the CFAR detectors’ own statistical assumptions to force consistently high false alarms (denial of service) for the malicious SU, while sparing genuine PUs by leaving their channels untouched. Unlike brute-force barrage jamming, our countermeasure targets the *sensing* layer of cognitive radio networks and uses deterministic, protocol-synchronised sweeping to guarantee predictable, reliable enforcement—a critical requirement in public-safety and military deployments. While the algorithm-aware philosophy shares common ground with the intelligent jamming strategies of^19,20^, those works operate at the communication layer against frequency-hopping and dynamic spectrum access links, whereas our design exploits the statistical structure of the CFAR reference window to enforce administrative spectrum policy.

We acknowledge that adapting CFAR detection to the frequency domain for spectrum sensing has appeared in prior works^12,13^; we do not claim the basic frequency-domain reformulation as a novel contribution. The distinguishing advances of this work, summarised in Table 1, are :

1. **Comprehensive multi-CFAR, multi-jammer evaluation.** We compare five CFAR variants (CA, GO, SO, OS, Censored) and a fixed-threshold baseline across three jamming scenarios (barrage, sweep-FM, comb-sweep) in a frequency-domain CR sensing framework—a breadth of analysis not found in prior work.
2. **Security paradox and algorithm-aware counter-access.** We identify that CFAR’s interference robustness paradoxically enables malicious SUs to evade administrative control, then resolve this by introducing a comb-sweep jammer that exploits CFAR reference-window statistics to drive $P_{fa} \rightarrow 1.0$ across all mean-level variants within a 4 dB JSR plateau ($-5$ to $-1$ dB) while preserving $P_d$ for genuine PUs. Simulations across varied CFAR window configurations confirm that no parameter choice grants the adversary an evasion advantage.

**Table 1:** Comparison of related CFAR-based sensing and jamming studies.

Reference	CR Context	Freq. Domain	Five CFAR	Multi-Jammer	Security Paradox
^12^	Yes	No	No	No	No
^13^	Yes	No	No	No	No
^14^	No	No	Yes	No	No
^15^	No	No	Yes	No	No
^21^	Yes	No	No	Yes	No
**Our work**	**Yes**	**Yes**	**Yes**	**Yes**	**Yes**

The remainder of this paper is organized as follows. CR paradigms, spectrum sensing techniques and their associated challenges–including noise uncertainty and jamming–are reviewed in Section "Background and related work", along with an introduction to the CFAR concept. The signal models, CFAR detection process within our framework, and considerations for wideband sensing are then described in Section "Frequency-domain CFAR spectrum sensing". Our frequency-domain CFAR detector design is presented in Section "Resilience of frequency-domain CFAR to interference and security-oriented threats", which includes simulation results demonstrating robust spectrum sensing performance under various jamming scenarios (barrage and sweeping), with comprehensive performance comparisons between CFAR variants and fixed-threshold approaches. Section "Administrator-controlled counter-access via comb-sweep jamming" introduces the comb-sweep jamming countermeasure, detailing its signal structure and demonstrating through simulations how it effectively induces service denial in CFAR-based SUs while preserving PU communications. The paper concludes in Section "Conclusion and future work" with key insights and directions for future research.

## Background and related work

This section lays the foundation for our work by reviewing essential concepts in CR, including its paradigms, spectrum sensing techniques, challenges from noise uncertainty and jamming attacks. We emphasize the interweave paradigm and derive equations for key elements to contextualize our contributions. These discussions highlight the need for robust, adaptive sensing in dynamic environments.

### Cognitive radio paradigms

CR networks operate under three primary paradigms: underlay, overlay, and interweave^22^. Each approach defines a different set of rules for how SUs can share spectrum with PUs. The underlay paradigm allows SUs to transmit concurrently with PUs as long as the interference they cause at the primary receiver is below a predefined threshold. This is typically achieved using spread spectrum techniques or by strictly limiting the SU’s transmit power^23^. A practical example of underlay systems includes ultra-wideband (UWB) devices that operate at very low power levels across wide frequency bands. The underlay model provides continuous, albeit low-power, access, but it requires precise power control and knowledge of channel conditions to avoid violating the interference threshold^24^. The overlay paradigm is the most complex approach. In this model, SUs possess extensive prior knowledge of the PU’s signal, which they can use to assist the primary network’s transmissions. For example, an SU might act as a cooperative relay to improve the PU’s signal quality while simultaneously using the channel for its own communication^25^. This paradigm can improve the overall efficiency and performance of both networks, but it requires close cooperation and shared information, raising significant security and privacy concerns^26^. The interweave paradigm, also known as opportunistic spectrum access, is the central focus of this paper. This model is predicated on the discovery that large portions of the licensed spectrum are often unused at a given time or location, a phenomenon referred to as ”spectrum holes” or ”white spaces^22^. A prominent real-world example of interweave systems is TV white space devices that opportunistically utilize unused television broadcast frequencies. In the interweave model, SUs must first perform spectrum sensing to detect the absence of a PU. If a spectrum hole is identified, the SU can transmit at full power, maximizing its throughput and efficiency. This mode of operation offers the greatest potential for significant improvement in spectrum utilization, but it is critically dependent on the accuracy and reliability of the spectrum sensing mechanism. A robust, low-latency spectrum sensing^27^ capability is the single most important technical requirement for a successful interweave system. It prevents harmful interference to PUs while maximizing opportunities for SUs.

### Spectrum sensing types

Spectrum sensing in cognitive radio networks can be universally formulated as a binary hypothesis testing problem^28^, where the secondary user seeks to determine whether a licensed spectrum band is idle or occupied by a primary user (PU). Let $y_s(t)$ denote the received baseband signal at the secondary user, which, in the presence of thermal noise *n*(*t*) and possible hostile interference *j*(*t*), can be expressed as

1 $$\begin{aligned} {\left\{ \begin{array}{ll} \mathcal {H}_0: y_s(t) = n(t) + j(t), & \text {PU absent}, \\ \mathcal {H}_1: y_s(t) = s_p(t) + n(t) + j(t), & \text {PU present}, \end{array}\right. } \end{aligned}$$

where $s_p(t)$ is the PU’s transmitted signal.

Irrespective of the specific detection strategy employed, the sensing problem reduces to computing a test statistic $T(y_s)$ from the received samples and comparing it against a threshold $\gamma$:

2 $$\begin{aligned} T(y_s) \;\underset{\mathcal {H}_0}{\overset{\mathcal {H}_1}{\gtrless }}\; \gamma . \end{aligned}$$

Different spectrum sensing methods differ only in the construction of $T(y_s)$: for instance, energy detection uses the average received energy; cyclostationary detection exploits spectral correlation functions; eigenvalue-based methods rely on the statistical structure of the sample covariance matrix.

For energy detection the natural continuous-time statistic over an observation interval $[0,T_0]$ is

3 $$\begin{aligned} T_{\textrm{eng}} = \frac{1}{T_0}\int _{0}^{T_0} |y_s(t)|^2\,\textrm{d}t . \end{aligned}$$

In practice the receiver samples $y_s(t)$ at rate $F_s$ ($T_s=1/F_s$), producing $N=T_0 F_s$ samples $y[n]=y_s(nT_s)$. The corresponding discrete-time implementation is

4 $$\begin{aligned} T_{\textrm{eng}} \approx \frac{1}{N}\sum _{n=1}^{N} |y[n]|^2 , \end{aligned}$$

which we use in all simulations (sampling assumed at or above Nyquist).

Under the common assumption $n(t)\sim \mathcal{C}\mathcal{N}(0,\sigma ^2)$ with independent samples (and in the absence of a deterministic structured jammer), the scaled energy statistic has the following distributions:

5 $$\begin{aligned} \frac{2N T_{\textrm{eng}}}{\sigma ^2} \sim {\left\{ \begin{array}{ll} \chi ^2_{2N}, & \mathcal {H}_0,\\ \chi '^2_{2N}(\lambda ), & \mathcal {H}_1, \end{array}\right. } \end{aligned}$$

where $\chi ^2_{2N}$ denotes the central chi-square distribution with 2*N* degrees of freedom, $\chi '^2_{2N}(\lambda )$ the noncentral chi-square with noncentrality parameter $\lambda$, and

6 $$\begin{aligned} \lambda =\frac{2E_s}{\sigma ^2}, \qquad E_s=\sum _{n=1}^N |s_p[n]|^2 , \end{aligned}$$

with $s_p[n]$ denoting the discrete-time samples of the PU signal.

Under $\mathcal {H}_0$, the scaled test statistic follows a central chi-square distribution with 2*N* degrees of freedom. Thus, the false alarm probability is

7 $$\begin{aligned} P_{\textrm{FA}}&= \Pr \{T_{\textrm{eng}}\ge \gamma \mid \mathcal {H}_0\} \nonumber \\&= 1 - F_{\chi ^2_{2N}}\!\Big (\tfrac{2N\gamma }{\sigma ^2}\Big ) \nonumber \\&= \frac{\Gamma \!\Big (N,\,\tfrac{N\gamma }{\sigma ^2}\Big )}{\Gamma (N)}, \end{aligned}$$

where $\Gamma (a,x)=\int _x^\infty t^{a-1}e^{-t}\,dt$ is the upper incomplete Gamma function and $\Gamma (a)$ the Gamma function.

For large *N*, a Gaussian central limit theorem approximation is often used:

8 $$\begin{aligned} P_{\textrm{FA}} \approx Q\!\Bigg (\frac{\gamma -\sigma ^2}{\sigma ^2/\sqrt{N}}\Bigg ), \end{aligned}$$

where $Q(x)=\tfrac{1}{\sqrt{2\pi }}\int _x^\infty e^{-t^2/2}\,dt$ is the Gaussian tail function. The corresponding detection probability under $\mathcal {H}_1$ is

9 $$\begin{aligned} P_{\textrm{D}}=\Pr \{T_{\textrm{eng}}\ge \gamma \mid \mathcal {H}_1\} =1-F_{\chi '^2_{2N}(\lambda )}\!\Big (\tfrac{2N\gamma }{\sigma ^2}\Big ), \end{aligned}$$

where $F_{\chi ^2}$ and $F_{\chi '^2}$ denote the CDFs of the central and noncentral chi-square distributions, respectively, and $Q(\cdot ,\cdot )$ the regularized upper incomplete Gamma. For large *N* a Gaussian central limit theorem approximation $T_{\textrm{eng}}\!\overset{\textrm{approx}}{\sim }\mathcal {N}(\mu _i,\sigma _i^2)$, $i\in \{0,1\}$, is often used for quick threshold design; exact chi-square/noncentral chi-square forms are used when precision is required.

The fundamental weakness of energy detection method is its reliance on accurate estimation of the background noise power. A slight deviation in this estimation can cause significant deviation from the desired false alarm rate, creating a performance floor known as the ”SNR wall” below which reliable detection is impossible^29^. Matched filter (MF) detection is the optimum detector for known signals in additive white Gaussian noise (AWGN) environments^30^. It operates by correlating the received signal with a perfectly known replica of the primary user’s (PU) waveform, thereby maximizing the output signal-to-noise ratio (SNR). The discrete-time test statistic over *N* received samples is

10 $$\begin{aligned} T_{\textrm{MF}} = \Re \left\{ \sum _{n=1}^{N} y[n]\, s_p^*[n] \right\} , \end{aligned}$$

where $s_p[n]$ is the known PU signal sequence and *y*[*n*] the received samples.

Assuming $n[n]\!\sim \!\mathcal{C}\mathcal{N}(0,\sigma ^2)$, the matched filter statistic follows a Gaussian distribution:

11 $$\begin{aligned} T_{\textrm{MF}} \sim {\left\{ \begin{array}{ll} \mathcal {N}(0,\,\tfrac{1}{2}N\sigma ^2), & \mathcal {H}_0, \\ \mathcal {N}(E_s,\,\tfrac{1}{2}N\sigma ^2), & \mathcal {H}_1, \end{array}\right. } \end{aligned}$$

Accordingly, the detection and false alarm probabilities are

12 $$\begin{aligned} P_{\textrm{FA}}=Q\!\left( \frac{\gamma }{\sqrt{\tfrac{1}{2}N\sigma ^2}}\right) , \quad P_{\textrm{D}}=Q\!\left( \frac{\gamma -E_s}{\sqrt{\tfrac{1}{2}N\sigma ^2}}\right) , \end{aligned}$$

with $Q(\cdot )$ denoting the Gaussian tail probability. While the MF achieves the best possible detection performance under AWGN, its practicality in cognitive radio networks is limited. It requires exact prior knowledge of the PU waveform (modulation, pulse shaping, synchronization, etc.), which is rarely available in opportunistic access settings. Thus, MF detection is best viewed as a theoretical performance upper bound rather than a deployable spectrum sensing strategy^4^.

Cyclostationary feature detection (CFD) exploits periodic statistical properties of communication signals, such as cyclic variations in autocorrelation, which are absent in stationary noise^31^. In discrete time, the cyclic autocorrelation is

13 $$\begin{aligned} R_y^\alpha [\tau ] = \lim _{N\rightarrow \infty } \frac{1}{N}\sum _{n=0}^{N-1} y[n]\,y^*[n-\tau ] \, e^{-j2\pi \alpha n}, \end{aligned}$$

and its Fourier transform yields the spectral correlation density (SCD). The test statistic is taken as the SCD magnitude at known cyclic frequencies:

14 $$\begin{aligned} T_{\textrm{CFD}} = \max _{\alpha ,f} |S_y^\alpha [f]| \;\underset{\mathcal {H}_0}{\overset{\mathcal {H}_1}{\gtrless }}\; \gamma . \end{aligned}$$

Under $\mathcal {H}_0$, noise yields $S_y^\alpha [f]\approx 0$, while under $\mathcal {H}_1$ the PU signal produces nonzero components at specific $\alpha$. This method is robust to noise uncertainty and effective at low SNRs, but requires high computational effort and long observation times^32^.

Eigenvalue-based detection is a blind sensing technique that exploits the statistical structure of the sample covariance matrix of the received signal^33^. Let $\textbf{y}=[y[1],\dots ,y[N]]^T$ denote *N* received samples and $\textbf{R}=\tfrac{1}{N}\textbf{y}\textbf{y}^H$ the estimated covariance matrix. In the noise-only case ($\mathcal {H}_0$), the eigenvalues of $\textbf{R}$ follow a predictable distribution, while the presence of a PU signal ($\mathcal {H}_1$) distorts this distribution, typically increasing the largest eigenvalues. A widely used test statistic is the ratio between the maximum eigenvalue and the average eigenvalue,

15 $$\begin{aligned} T_{\textrm{EVD}} = \frac{\lambda _{\max }}{\tfrac{1}{M}\sum _{i=1}^M \lambda _i}, \end{aligned}$$

where $\lambda _{\max }$ is the maximum eigenvalue, $\{\lambda _i\}_{i=1}^M$ are all eigenvalues of $\textbf{R}$, and *M* is its dimension. This method is inherently robust to noise power uncertainty, since the decision relies on eigenvalue ratios rather than absolute energy levels. However, as with cyclostationary detection, the need to compute and decompose the covariance matrix makes it computationally demanding^34^.

### Noise uncertainty and jamming attacks

Noise uncertainty is a pervasive challenge in spectrum sensing, referring to the deviation of the true noise power from its assumed value^29,35^. Such deviations may arise from temperature variations, atmospheric effects, hardware imperfections, or in-band interference sources. According to the central limit theorem, the aggregate contribution of multiple independent interferers can cause the effective noise to deviate from the Gaussian assumption, complicating estimation and leading to degraded sensing performance. Noise uncertainty is commonly modeled by a mismatch factor

$$\rho = \frac{\sigma _w^2}{\hat{\sigma }_w^2},$$

where $\sigma _w^2$ is the true noise variance and $\hat{\sigma }_w^2$ its estimate. For a fixed-threshold energy detector, even small errors in $\rho$ cause the actual false alarm probability to deviate from its nominal design value, a limitation that manifests as the so-called SNR wall^36^.

Using the Gaussian approximation of the energy detector statistic, the false alarm probability under $\mathcal {H}_0$ can be expressed as

16 $$\begin{aligned} P_{\textrm{FA}} \approx Q\!\Bigg (\frac{\gamma - \rho }{\rho /\sqrt{N}}\Bigg ), \end{aligned}$$

where $Q(x)=\tfrac{1}{\sqrt{2\pi }}\int _x^\infty e^{-t^2/2}\,dt$ is the Gaussian tail function, $\gamma$ is the normalized detection threshold, and *N* is the number of collected samples. This formulation highlights how noise uncertainty shifts the effective threshold, potentially leading to severe performance degradation in practice.

Beyond intrinsic uncertainty, spectrum sensing performance is further degraded under adversarial interference. Jamming attacks deliberately inject disruptive signals to impair spectrum sensing and communication. Comprehensive surveys summarize their forms and countermeasures in wireless networks^37^. Conventional jamming attacks can be broadly classified according to their spectral characteristics^38^:

1. **Barrage Jamming:** Transmits wideband noise across the entire spectrum of interest. The jamming signal can be modeled as additive white Gaussian noise (AWGN) with variance $\sigma _j^2$, i.e., $j(t)\sim \mathcal {N}(0,\sigma _j^2)$. While simple and effective over broad bands, it requires high power and is easily detected^39^.
2. **Spot Jamming:** Concentrates power in a narrow frequency band using band-limited noise. If $n_b(t)$ is white noise band-limited to bandwidth $B_j$ around $f_j$, the jammer is
  17 $$\begin{aligned} j(t)=\sqrt{P_j}\,n_b(t), \end{aligned}$$
  with power spectral density
  18 $$\begin{aligned} S_j(f) = \frac{P_j}{B_j}, \quad |f-f_j|\le \tfrac{B_j}{2}. \end{aligned}$$
  This approach is power-efficient and highly disruptive to specific channels, but requires knowledge of the victim frequency and is mitigated by frequency agility^40^.
3. **Sweep Spot Jamming:** Employs a narrowband jammer that sweeps across a range of frequencies over time. For a linear sweep across $\Delta f$ in period $T_s$, the instantaneous frequency is
  19 $$\begin{aligned} f_c(t)=f_s+\frac{\Delta f}{T_s}(t \bmod T_s), \end{aligned}$$
  where $f_s$ is the starting frequency. Sweep spot jamming is more efficient than barrage jamming and can disrupt multiple channels sequentially, but cannot block all channels simultaneously and is less effective against rapid frequency hopping^21^.

These forms of interference present major obstacles for conventional sensing schemes, especially fixed-threshold energy detectors, which assume stationary noise statistics. Their limitations motivate the use of adaptive techniques such as CFAR detection, which dynamically adjusts thresholds in response to varying interference conditions, as discussed in the following sections. However, as Section "Resilience of frequency-domain CFAR to interference and security-oriented threats" demonstrates, this same adaptivity introduces a security paradox: a CFAR-equipped adversary can resist even deliberate jamming attempts. This observation ultimately motivates the design of a more targeted, algorithm-aware countermeasure—the comb-sweep jammer introduced in Section "Administrator-controlled counter-access via comb-sweep jamming"—which moves beyond conventional spectral interference to exploit the CFAR mechanism’s own statistical assumptions.

### Channel impairments and fading models

The binary hypothesis framework in (1) implicitly corresponds to the AWGN channel, where the received signal contains only additive noise and possible jamming. In practical wireless environments, however, fading alters the propagation of PU signals and directly affects their communication reliability, particularly in terms of bit error rate (BER).

In a **flat Rayleigh fading** channel, all frequency components of the PU signal experience the same attenuation and phase shift. The received signal under $\mathcal {H}_1$ becomes

20 $$\begin{aligned} y_s(t) = h s_p(t) + n(t) + j(t), \end{aligned}$$

where $h=\alpha e^{j\phi }$ is the complex channel gain with Rayleigh-distributed magnitude $\alpha$ and uniformly distributed phase $\phi$. The randomness of *h* causes fluctuations in the instantaneous SNR, leading to a higher BER compared to the AWGN case.

In a **frequency-selective fading** channel, different spectral components of $s_p(t)$ undergo different attenuations and delays, yielding

21 $$\begin{aligned} y_s(t) = \sum _{l=0}^{L-1} h_l s_p(t-\tau _l) + n(t) + j(t), \end{aligned}$$

where $\{h_l,\tau _l\}$ denote the multipath gains and delays. The superposition of delayed replicas results in intersymbol interference (ISI), which further degrades the BER performance of the PU.

These fading models emphasize that PU communication reliability is significantly influenced by channel impairments. While our focus remains on spectrum sensing under uncertainty and jamming, it is important to recognize that the overall CRN performance is also tightly coupled with PU link reliability under realistic propagation conditions.

## Frequency-domain CFAR spectrum sensing

The concept of CFAR detection originated in radar applications, where the task is to search for targets across range and Doppler bins. The decision statistic is evaluated as *T*(*r*, *d*) for each range–Doppler cell, and compared to an adaptive threshold to maintain a constant false alarm rate. In cognitive radio, the sensing problem is naturally reformulated in the frequency domain: instead of range–Doppler cells, the receiver monitors spectral bins $\{f_k\}$ and evaluates statistics $T(f_k)$ to identify the presence of PUs or adversarial interference. Classical energy detection with a fixed threshold $\gamma$ offers low complexity but is highly sensitive to noise uncertainty, while matched filtering achieves optimal performance only when the PU waveform $s_p(t)$ is perfectly known, which is rarely feasible in opportunistic access. Advanced approaches such as cyclostationary detection or eigenvalue-based methods improve robustness but at prohibitive computational cost. This gap motivates the need for a frequency-domain CFAR reformulation: a blind, scalable, and computationally efficient technique that adapts classical CFAR principles to the challenges of wideband spectrum sensing under interference and jamming.

To structure our discussion, we first highlight the vulnerability of fixed-threshold detectors to low-power interference. We then reformulate the CFAR principle in the frequency domain and present the resulting detection framework. Simulation results are provided to evaluate performance under diverse conditions, and finally, we discuss the security paradox wherein robustness itself may be exploited as a vulnerability.

### Fixed-threshold vulnerability to low-power interference

**Fig. 1:**
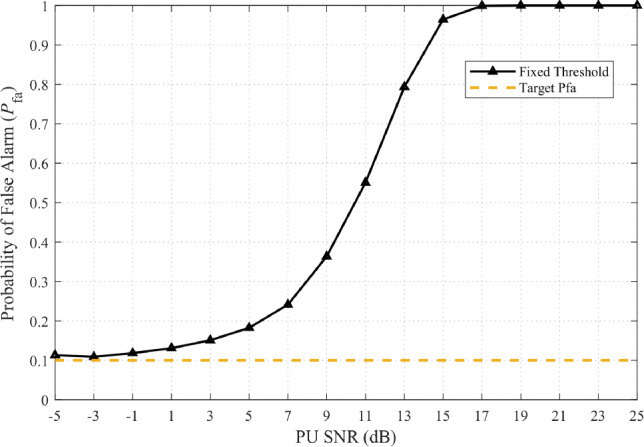
False alarm probability escalation under broadband interference at ISR = −20 dB demonstrating fixed-threshold vulnerability.

Fixed-threshold energy detection is attractive for secondary users (SUs) due to its low complexity, yet it is inherently fragile in non-stationary environments. Even weak broadband interference can destabilize the false-alarm rate, undermining reliable spectrum access (Fig. 1).

The issue stems from the static threshold design: a fixed $\gamma$ is chosen to satisfy the Neyman–Pearson constraint under nominal noise,

22 $$\begin{aligned} \mathbb {P}\!\left[ T>\gamma \,\big |\, \mathcal {H}_0 \right] = \alpha , \end{aligned}$$

but once an interference component *j*(*t*) elevates the effective background power, the actual false-alarm probability grows far beyond the target,

23 $$\begin{aligned} \mathbb {P}\!\left[ T>\gamma \,\big |\, \mathcal {H}_0+j(t) \right] \gg \alpha . \end{aligned}$$

This “false-alarm inflation” phenomenon is a central weakness of fixed-threshold sensing.

**Fig. 2:**
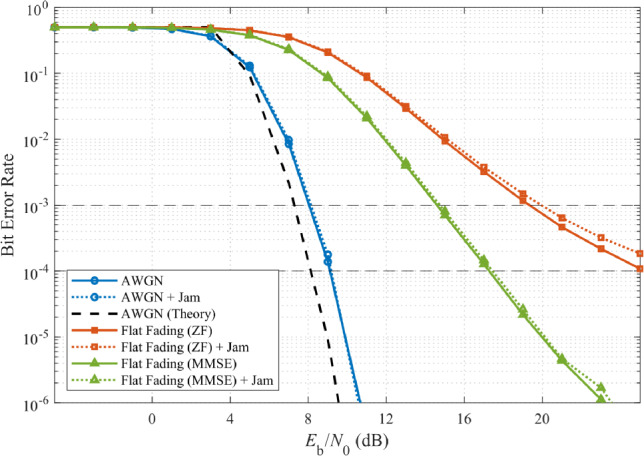
P25 PU BER performance under broadband interference at ISR = −20 dB showing communication resilience.

A representative case study clarifies the disparity between PU communication robustness and SU sensing fragility. Consider a P25 public-safety system operating with PU power at $-90$ dBm, receiver sensitivity of $-100$ dBm, and broadband interference at $-110$ dBm (interference-to-signal ratio ISR = $-20$ dB). For the PU, such interference is negligible: Fig. 2 shows BER performance remains nearly identical to the AWGN benchmark, with only a slight deviation due to the root-raised-cosine pulse shaping filter^41^. Robust modulation and coding absorb the weak interference without service degradation. This overall resilience stems from the robust modulation and coding schemes inherent in P25 telecommunication standard as shown in Table 2.

**Table 2:** P25 PU System Parameters.

Parameter	Value
Modulation	$\pi /4$-DQPSK
Modulation Order (*M*)	4
Symbol Rate ($R_s$)	4800 symbols/s
Convolutional Code Rate	1/2
Pulse Shaping Filter	RRC, $\alpha = 0.2$
Filter Span	6 symbols
Oversampling Factor	8
Sampling Rate ($F_s$)	38.4 kHz

In stark contrast, SU spectrum sensing collapses under the same conditions. Fig. 1 demonstrates that the realized false-alarm probability of a fixed-threshold detector rapidly escalates toward unity as SNR increases, effectively creating a denial-of-service for the SU. Although the PU link remains fully operational, the SU is locked out of spectrum access.

This discrepancy underscores a critical vulnerability: fixed-threshold detectors, though simple, cannot ensure reliable operation in dynamic environments with low-power jamming or interference. The gap between (22) and (23) motivates adaptive thresholding strategies, such as CFAR, which restore the Neyman–Pearson false-alarm control by continuously normalizing against interference power.

### Frequency-domain CFAR reformulation

**Fig. 3:**
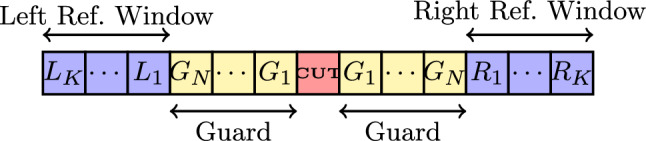
Generalized CFAR window structure in the frequency domain. Each bin corresponds to the magnitude of one spectral component of the SU’s received signal.

The CFAR process employs a sliding window that scans the received signal, evaluating one data point or frequency bin at a time. See Fig. 3, where the window consists of three main components:

1. **Cell Under Test (CUT):** The specific data point or frequency bin being assessed for the presence of a signal.
2. **Guard Cells:** Cells immediately adjacent to the CUT, excluded from noise estimation to avoid contamination by signal energy leakage from the CUT in the form of spectral sidelobes.
3. **Reference Cells:** Cells on either side of the guard cells, used to estimate the local noise and interference floor.

Each cell in the window represents the absolute value of the signal amplitude. The window consists of the CUT, $y_0$, surrounded by $N_g$ guard cells and *K* reference cells on each side. The reference cells are divided into left and right segments, denoted as $L_i$ and $R_i$ respectively, where $L_i = |y_{-(N_g+i)}|$ for $i = 1, 2, \ldots , K$ (left reference cells) and $R_i = |y_{N_g+i}|$ for $i = 1, 2, \ldots , K$ (right reference cells). Based on the statistical properties of the reference cells, various CFAR variants compute the noise estimate *Z*, which sets the adaptive threshold $T_{\text {CFAR}} = \alpha \cdot Z$, where $\alpha$ is a scaling factor determined by the desired false alarm probability.

We reformulate this framework for cognitive radio by applying it in the frequency domain. Rather than sliding in time or range (as in radar), the CFAR window traverses across frequency bins obtained via spectral analysis of the SU’s received signal. A PU manifests as a localized spectral spike, while the surrounding bins capture background noise and interference statistics. This frequency-domain adaptation allows the SU to normalize its threshold against local interference conditions, enabling robust spectrum sensing in dynamic wideband environments.

#### Frequency-domain processing and CFAR cellization

The secondary user periodically acquires *N* samples of the received signal *y*(*t*) and computes their *N*-point FFT. Denote the coefficient at bin *k* by *Y*[*k*], and form the periodogram

24 $$\begin{aligned} P[k] = |Y[k]|^2, \qquad k=0,1,\ldots ,N-1 , \end{aligned}$$

with frequency resolution $B_{\textrm{FFT}} = F_s/N$.

Since primary user (PU) signals typically occupy finite bandwidths, the spectrum is partitioned into non-overlapping *cells*, each aggregating *M* consecutive FFT bins. The cell size is chosen according to the PU bandwidth $B_{\text {PU}}$ relative to the FFT resolution:

25 $$\begin{aligned} M = \Big \lceil \frac{B_{\text {PU}}}{B_{\text {FFT}}} \Big \rceil . \end{aligned}$$

Cells are indexed as $c=1,2,\ldots ,C$, where $C=\lfloor N/M \rfloor$. The first bin of cell *c* is

26 $$\begin{aligned} k_c = (c-1)M , \end{aligned}$$

so that cell *c* spans bins $k_c, k_c+1, \ldots , k_c+M-1$. The average power in cell *c* is defined as

27 $$\begin{aligned} \bar{P}_c = \frac{1}{M} \sum _{k=k_c}^{k_c+M-1} P_k . \end{aligned}$$

which serves as the test statistic for CFAR processing at cell *c*.

Building upon this spectral representation, a CFAR sliding window is employed in the frequency domain, as illustrated in Fig. 3. The window operates at the cell level for a given cell $c_0$ serving as the CUT. The CFAR window forms a vector of length $2N_g + 2K + 1$ cells, consisting of the CUT at $c_0$, $2N_g$ guard cells ($c_0 \pm 1$ to $c_0 \pm N_g$), and 2*K* reference cells ($c_0 \pm (N_g + 1)$ to $c_0 \pm (N_g + K)$).

**Fig. 4:**
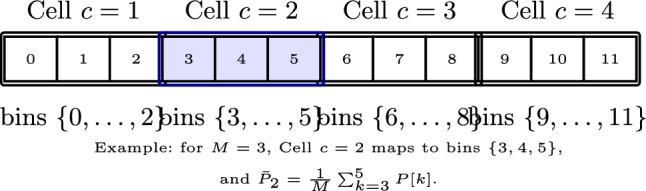
Mapping from FFT bins to CFAR cells ($M=3$). Each cell aggregates $M$ consecutive bins. The highlighted cell ($c=2$) shows the averaged power computation.

As the CFAR window slides across the spectrum by incrementing $c_0$, subchannel-by-subchannel analysis is enabled. For enhanced granularity against narrowband interference, the cells may overlap (e.g., with a sliding step size less than *m* bins), ensuring the detector remains resilient to heterogeneous narrowband interference that could otherwise impair conventional wideband energy detection. To illustrate this formulation using the actual simulation parameters adopted throughout this work, for $N=1024$ FFT points at $F_s=2.4576$ MHz the resolution is $B_{\textrm{FFT}}=F_s/N\approx 2.4$ kHz. A P25 channel of $B_{\textrm{PU}}=12.5$ kHz yields $M=\lceil 12.5/2.4 \rceil = 6$ bins per cell. Thus cell $c=2$ corresponds to bins $\{6,7,8,9,10,11\}$ and its average power is $\bar{P}_2 = \tfrac{1}{6}\sum _{k=6}^{11}P[k]$ (see Fig. 4, which illustrates the general principle with $M=3$; the actual simulation uses $M=6$).

#### CFAR variants in frequency domain

The CFAR detection procedure for each cell $c_0$ involves three sequential steps: noise estimation, threshold setting, and decision making. Using the average power per cell defined in (27), we construct reference sets for the CUT at index $c_0$ as

28 $$\begin{aligned} \mathcal {R}_L(c_0)&= \{\,c_0-(N_g+N_r),\ldots ,c_0-(N_g+1)\,\}, \end{aligned}$$

29 $$\begin{aligned} \mathcal {R}_R(c_0)&= \{\,c_0+(N_g+1),\ldots ,c_0+(N_g+N_r)\,\}, \end{aligned}$$

30 $$\begin{aligned} \mathcal {R}(c_0)&= \mathcal {R}_L(c_0) \cup \mathcal {R}_R(c_0), \qquad |\mathcal {R}(c_0)|=2N_r . \end{aligned}$$

Noise estimates for different CFAR variants are then defined as follows:

**CA-CFAR:** The average power across all reference cells,

31 $$\begin{aligned} Z_{CA} = \frac{1}{|\mathcal {R}(c_0)|} \sum _{j \in \mathcal {R}(c_0)} \bar{P}_j . \end{aligned}$$

**GO-CFAR and SO-CFAR:** The maximum or minimum between left and right reference averages,

32 $$\begin{aligned} Z_{GO} = \max \!\Bigg ( \frac{1}{N_r}\sum _{j \in \mathcal {R}_L(c_0)} \bar{P}_j ,\; \frac{1}{N_r}\sum _{j \in \mathcal {R}_R(c_0)} \bar{P}_j \Bigg ),\end{aligned}$$

33 $$\begin{aligned} Z_{SO} = \min \!\Bigg ( \frac{1}{N_r}\sum _{j \in \mathcal {R}_L(c_0)} \bar{P}_j ,\; \frac{1}{N_r}\sum _{j \in \mathcal {R}_R(c_0)} \bar{P}_j \Bigg ). \end{aligned}$$

**OS-CFAR:** This approach selects the *r*-th order statistic of the sorted reference cell powers, extending the time-domain OS-CFAR principle to the frequency domain:

34 $$\begin{aligned} Z_{OS} = \bar{P}_{(r)}, \end{aligned}$$

where $\bar{P}_{(r)}$ denotes the *r*-th smallest average power among the $|\mathcal {R}(c_0)|$ reference cells. Following common practice in radar CFAR design, selecting $r \approx 60\%$–$80\%$ of the reference window size provides a good trade-off between robustness to interference and sensitivity to weak signals^11^. In our setup, with $|\mathcal {R}(c_0)| = 16$ reference cells, we set $r = 12$.

**Censored-CFAR:** This variant discards the $M_c$ largest reference cell powers prior to averaging, thereby suppressing the impact of strong narrowband interferers:

35 $$\begin{aligned} Z_C = \frac{1}{|\mathcal {R}(c_0)| - M_c} \sum _{i=1}^{|\mathcal {R}(c_0)| - M_c} \bar{P}_{(i)}, \end{aligned}$$

where $\bar{P}_{(i)}$ denotes the *i*-th smallest ordered reference power. Literature suggests censoring a small fraction (typically 15–25%) of the highest-power reference cells to maintain robustness without excessive sensitivity loss^11^. We adopt $M_c = 4$, corresponding to censoring 25% of the reference cells.

Once the noise estimate is computed, we denote it generically as $Z_{c_0}$, where

36 $$\begin{aligned} Z_{c_0} \in \{ Z_{CA}, \; Z_{GO}, \; Z_{SO}, \; Z_{OS}, \; Z_{C} \}, \end{aligned}$$

depending on the chosen CFAR variant. The adaptive detection threshold for the CUT at index $c_0$ is then expressed as

37 $$\begin{aligned} \lambda _{c_0} = \alpha \, Z_{c_0}, \end{aligned}$$

where $\alpha$ is the scaling constant determined by the desired false-alarm probability $P_{\textrm{FA}}$ and the number of reference cells. Importantly, $\alpha$ remains fixed for a given configuration, independent of the actual noise or interference level.

The final decision rule is therefore

38 $$\begin{aligned} \delta (c_0) = {\left\{ \begin{array}{ll} \mathcal {H}_1, & \bar{P}_{c_0}> \lambda _{c_0}, \\ \mathcal {H}_0, & \bar{P}_{c_0} \le \lambda _{c_0}, \end{array}\right. } \end{aligned}$$

where $\bar{P}_{c_0}$ is the average cell power defined in (27). Applying this procedure across all candidate cells yields a frequency-domain occupancy map that adapts locally to heterogeneous interference conditions, unlike static threshold detectors.

## Resilience of frequency-domain CFAR to interference and security-oriented threats

Having established the theoretical formulation and implementation framework of the proposed frequency-domain CFAR detector, we now turn to its empirical validation and security-oriented assessment. This section presents a comprehensive performance evaluation under diverse interference environments representative of realistic cognitive radio (CR) operation. The analysis quantifies how adaptive thresholding in the spectral domain enhances sensing robustness against both stationary and time-varying interference sources. Furthermore, beyond communication performance, we uncover a critical cybersecurity dimension–demonstrating that the same robustness which protects secondary users (SUs) from interference can paradoxically undermine administrative spectrum control, motivating the development of intelligent counter-access mechanisms in subsequent sections.

### Simulation setup and parameters

Monte Carlo simulations were conducted using APCO Project 25 (P25) waveforms for primary users (PUs) and OFDMA-based secondary users (SUs). The CFAR configuration parameters are summarized in Table 3, while system and channel settings are provided in Table 4. Unless otherwise stated, all simulations were performed over an additive white Gaussian noise (AWGN) channel with noise uncertainty (NU) of 1 dB and interference-to-signal ratio (ISR) adjusted per scenario. Equal average interference power was maintained across all cases to ensure fair comparison.

**Table 3:** Frequency-Domain CFAR Configuration Parameters.

Parameter	Value
Total Subchannels	50
Reference Window Size	[6–20] cells
Guard Cells	[0–2] per side
Target False Alarm Rate	0.1
Detection Domain	Frequency
Window Type	Sliding
OS Order (*k*)	75% of Ref. window
Censored Cells ($M_c$)	25% of Ref. window
Monte Carlo Trials	10,000

**Table 4:** Spectrum Sensing Simulation Parameters.

Parameter	Value
Sampling Rate ($F_s$)	2.4576 MHz
FFT Size (*N*)	1024
Observation Duration ($T_0$)	$N/F_s = 416.67\;\mu$s
Freq. Resolution ($B_\textrm{FFT}$)	$F_s/N \approx 2.4$ kHz
Subchannel Bandwidth ($B_\textrm{ch}$)	12.5 kHz
Bins per Cell (*M*)	$\lceil B_\textrm{ch}/B_\textrm{FFT}\rceil = 6$
Channel Models	AWGN
Noise Uncertainty (NU)	1 dB
Monte Carlo Trials	10,000

### Performance under wideband interference

Under wideband interference, the disturbance power is distributed approximately uniformly across all frequencies, effectively elevating the background noise floor. A fixed threshold $\gamma$ calibrated for quiet conditions thus misrepresents $\mathcal {H}_0$, causing inflation in the false-alarm probability. By contrast, the adaptive CFAR detector continuously updates its decision threshold according to the local noise estimate $Z_{c_0}$:

39 $$\begin{aligned} \gamma _{c_0} = \alpha _{\textrm{CFAR}} \, Z_{c_0},\quad \Pr \!\left\{ T_{c_0}> \gamma _{c_0} \mid \mathcal {H}_0 \right\} = P_{\textrm{FA}}^{*}, \end{aligned}$$

and $P_{\textrm{FA}}^{*}$ is the desired false-alarm probability target. Because the estimate $Z_{c_0}$ tracks the interference floor, the adaptive threshold “rides” the interference-induced elevation in background power, maintaining constant false-alarm rate (CFAR property).

**Fig. 5:**
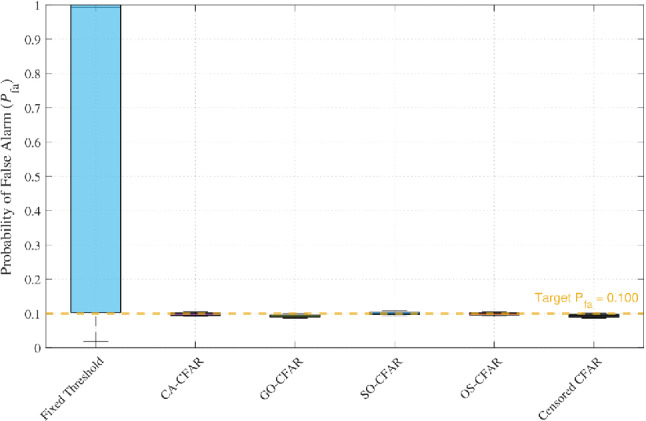
False-alarm probability comparison under wideband interference (JSR = –10 dB, NU = 1 dB).

**Fig. 6:**
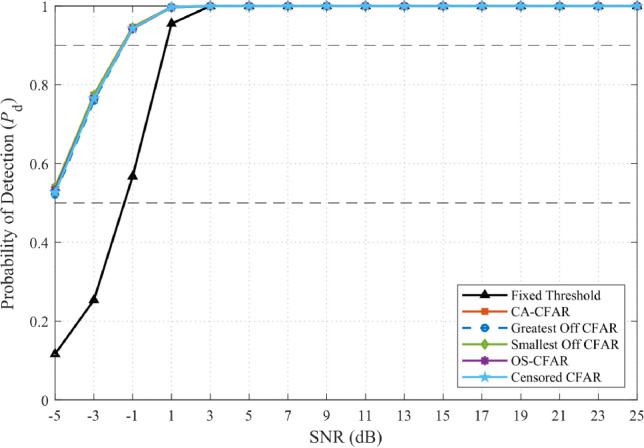
Detection probability under wideband interference (JSR = –10 dB, NU = 1 dB).

Simulations with ISR = −10 dB confirmed this behavior. As illustrated in Fig. 5, fixed-threshold detectors exhibit catastrophic false-alarm inflation (approaching unity at SNR $\ge$ 10 dB), while all CFAR variants maintain $P_{\textrm{FA}} \approx 0.1$. Figure 6 further demonstrates that adaptive thresholding not only preserves $P_{\textrm{FA}}$ but also sustains high detection probability $P_{\textrm{D}}$, confirming the robustness of frequency-domain CFAR against homogeneous wideband interference.

### Performance under sweep-FM jamming

More challenging interference conditions arise when a jammer occupies only a fraction of the spectrum at a time, as in sweep-modulated frequency (FM) interference.

To model a swept (linear) FM jammer whose instantaneous center frequency follows the previously introduced law

40 $$\begin{aligned} f_c(t)=f_s+\frac{\Delta f}{T_s}\big (t \bmod T_s\big ), \end{aligned}$$

we represent the continuous-time swept-FM complex baseband waveform by its instantaneous phase:

41 $$\begin{aligned}&s_{\textrm{sw}}(t) = A_j \exp \!\big ( j\,\phi (t)\big ),\nonumber \\&\phi (t) \;=\; 2\pi \int _{0}^{t} f_c(\tau )\,\textrm{d}\tau + \phi _0, \end{aligned}$$

where $A_j$ is the jammer amplitude and $\phi _0$ an initial phase. Substituting (40) and integrating over one sweep interval (denote the sweep period in subsequent expressions by $T_{\textrm{sw}} \equiv T_s$) yields, for the fractional time within the current sweep $u(t)\!=\!(t \bmod T_{\textrm{sw}})\in [0,T_{\textrm{sw}})$,

42 $$\begin{aligned} \phi (t) \;=\; 2\pi \!\left( f_s t + \frac{\Delta f}{2T_{\textrm{sw}}} u(t)^2 \right) + \phi _0. \end{aligned}$$

For discrete-time simulation with sampling frequency $F_s$ and sampling interval $T_{\textrm{sam}}=1/F_s$, let $n$ be the sample index and $u_n = (nT_{\textrm{sam}} \bmod T_{\textrm{sw}})$. The sampled swept-FM waveform is

43 $$\begin{aligned} s_{\textrm{sw}}[n] = A_j \exp \!\bigg \{ j\,2\pi \!\Big ( f_s nT_{\textrm{sam}} + \frac{\Delta f}{2T_{\textrm{sw}}} u_n^2 \Big ) + j\phi _0 \bigg \}. \end{aligned}$$

This discrete-time expression is numerically stable and convenient to implement: compute $u_n$ as the fractional time within the sweep and evaluate the phase using (43).

When comparing narrowband or swept interference to a broadband (barrage) baseline, it is necessary to express the jammer strength relative to the power that would fall into a single victim channel under the barrage condition. Let $P_J$ denote the jammer’s total transmitted power, $B_{\textrm{barr}}$ the bandwidth of the barrage (reference) jammer, $B_{\textrm{occ}}$ the instantaneous occupied bandwidth of the narrowband or swept jammer, $B_{\textrm{ch}}$ the victim channel bandwidth, and $B_{\textrm{ovl}}$ the instantaneous overlap between the jammer footprint and the victim channel. Assuming approximately flat instantaneous PSD across the jammer footprint, the instantaneous jammer power incident on the victim channel is

44 $$\begin{aligned} P_{J,\text {chan}}^{\textrm{occ}} = P_J \frac{B_{\textrm{ovl}}}{B_{\textrm{occ}}}, \qquad P_{J,\text {chan}}^{\textrm{barr}} = P_J \frac{B_{\textrm{ch}}}{B_{\textrm{barr}}}. \end{aligned}$$

Hence, the instantaneous per-channel power advantage of the narrow or swept jammer relative to the barrage reference is

45 $$\begin{aligned} \Gamma = \frac{P_{J,\text {chan}}^{\textrm{occ}}}{P_{J,\text {chan}}^{\textrm{barr}}} = \frac{B_{\textrm{ovl}}}{B_{\textrm{occ}}}\, \frac{B_{\textrm{barr}}}{B_{\textrm{ch}}}. \end{aligned}$$

For time-varying (swept) jammers that dwell for $T_{\textrm{dwell}}$ on each subchannel within a sweep period $T_{\textrm{sw}}$, the duty factor $\rho = T_{\textrm{dwell}} / T_{\textrm{sw}}$ scales this advantage to a time-averaged value,

46 $$\begin{aligned} \Gamma _{\textrm{avg}} = \rho \,\Gamma . \end{aligned}$$

The effective per-channel JSR relative to the barrage baseline therefore becomes

47 $$\begin{aligned} \textrm{JSR}_{\textrm{chan}} = 10 \log _{10} (\Gamma _{\textrm{avg}}), \end{aligned}$$

capturing both the spectral-efficiency gain of narrowband interference and the reduced temporal occupancy due to sweeping. For example, with $B_{\textrm{barr}}=6~\text {MHz}$, $B_{\textrm{ch}}=12.5~\text {kHz}$, $B_{\textrm{occ}}=B_{\textrm{ovl}}=12.5~\text {kHz}$, and $\rho =0.1$, the instantaneous advantage is $\Gamma \approx 480$ (26.8 dB), yielding an average advantage $\Gamma _{\textrm{avg}} \approx 48$ (16.8 dB).

The sweep-FM scenarios used in our experiments are chosen to be directly tied to the SU sensing parameters (sampling rate and FFT size) so that dwell-time, sweep period and instantaneous spectral coverage are physically meaningful for the simulated detector. Let the SU sampling rate be $F_s$ and the number of acquired samples per sensing interval be *N*, so the capture (observation) duration is

48 $$\begin{aligned} T_0 = \frac{N}{F_s}. \end{aligned}$$

With $F_s = 2.4576\ \textrm{MHz}$ and $N=1024$ we obtain

$$T_0 = \frac{1024}{2.4576\times 10^6} \approx 416.67\ \mu \textrm{s},$$

which is the time window over which each FFT-based spectral estimate is computed.

Assume the total band is partitioned into $C=50$ subchannels of bandwidth $B_{\textrm{ch}}=12.5\ \textrm{kHz}$. For the purpose of mapping jammer behavior to the SU capture window we set the sweep period equal to the capture duration, $T_{\textrm{sw}}=T_0$, which makes $\rho$ directly interpretable as the fraction of the SU sensing window during which a given subchannel is jammed.

1. **Conservative (narrow-coverage) scenario.** We choose a dwell time computed from the subchannel reciprocal bandwidth (one symbol-equivalent interval) plus a small hardware switching overhead $t_{\textrm{sw}}$:
  $$T_{\textrm{dwell}} = \frac{1}{B_{\textrm{ch}}} + t_{\textrm{sw}}.$$
  With $B_{\textrm{ch}}=12.5\ \textrm{kHz}$ and $t_{\textrm{sw}}=0.2\ \mu \textrm{s}$ this yields
  $$T_{\textrm{dwell}} = 80\ \mu \textrm{s} + 0.2\ \mu \textrm{s} = 80.2\ \mu \textrm{s}.$$
  Within the capture duration $T_0\approx 416.67\ \mu \textrm{s}$ the jammer therefore covers approximately
  $$N_{\textrm{cov}} = \frac{T_0}{T_{\textrm{dwell}}} \approx \frac{416.67}{80.2} \approx 5.2$$
  subchannels per sweep cycle – i.e., roughly 5 subchannels, or 10% of the 50-channel band. The corresponding duty factor is $\rho \approx 80.2/416.67 \approx 0.192$.
2. **Aggressive (wide-coverage) scenario.** To emulate a rapid-sweep, high-coverage jammer we reduce the dwell time by a factor $\alpha _{\textrm{dwell}}=0.25$:
  $$T_{\textrm{dwell}}^{\mathrm {(agg)}} = \alpha _{\textrm{dwell}}\,T_{\textrm{dwell}}^{\mathrm {(cons)}} = 0.25\times 80.2\ \mu \textrm{s} \approx 20.05\ \mu \textrm{s}.$$
  With this reduced dwell, the number of subchannels covered in one capture is
  $$N_{\textrm{cov}}^{\mathrm {(agg)}} \approx \frac{416.67}{20.05} \approx 20.8,$$
  i.e., roughly 20 subchannels (40% of the 50-channel band). The duty factor per channel drops to $\rho \approx 20.05/416.67\approx 0.048$.

We assume the jammer maintains constant total transmitted power $P_J$ across scenarios (equal-total-power assumption used throughout our experiments). Under this constraint the instantaneous power delivered to each jammed subchannel scales inversely with the number of simultaneously affected subchannels. Hence, increasing coverage by a factor of roughly four (from $\approx 5$ to $\approx 20$ subchannels) reduces the instantaneous per-channel jamming power by a factor of four, i.e. by

$$10\log _{10}(4) \approx 6.02\ \textrm{dB},$$

which explains the statement that the aggressive sweep reduces per-channel jamming power by approximately 6 dB relative to the conservative sweep. Conversely, the conservative sweep concentrates the same total power onto fewer channels, producing a higher instantaneous JSR on the jammed subchannels. We note the following remarks on modelling assumptions

1. We set $T_{\textrm{sw}}=T_0$ to align the sweep period with the SU sensing interval; this choice simplifies the mapping between $\rho$, $N_{\textrm{cov}}$ and the SU’s observed energy per bin. If the jammer’s sweep period differs from $T_0$, $\rho$ should be computed accordingly.
2. The small switching overhead $t_{\textrm{sw}}$ accounts for practical hardware delays; its magnitude can be adjusted to reflect a given platform.
3. The equal-total-power assumption isolates the effect of spectral coverage (number of jammed channels) from total transmitted energy. If one wishes to simulate variable-power jammers, scale $P_J$ accordingly and recompute instantaneous per-channel JSR via the overlap expressions in Section "Resilience of frequency-domain CFAR to interference and security-oriented threats".

**Fig. 7:**
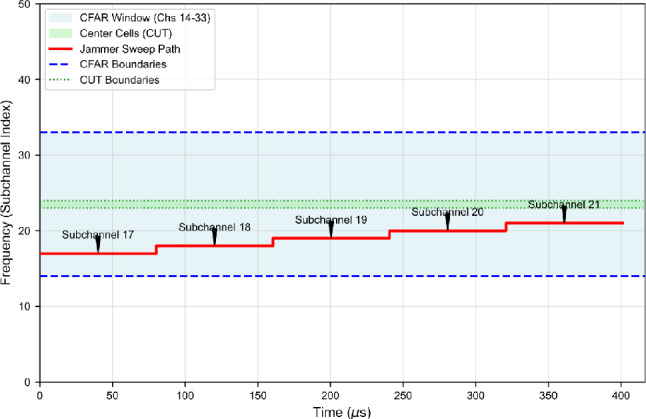
Time-frequency representation of narrow-sweep interference covering 5 of 50 subchannels.

**Fig. 8:**
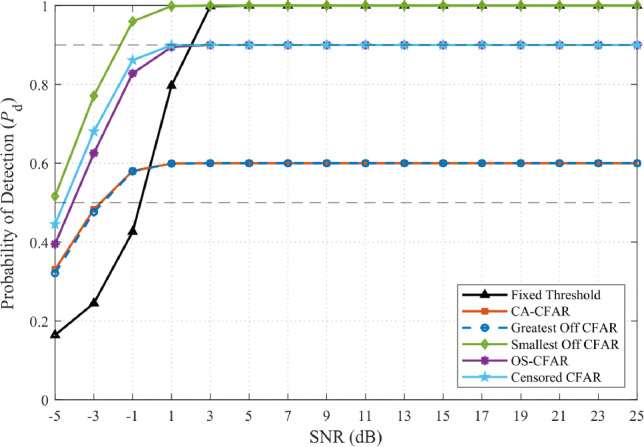
CFAR performance under narrow-sweep interference (JSR = 7 dB, NU = 1 dB).

**Fig. 9:**
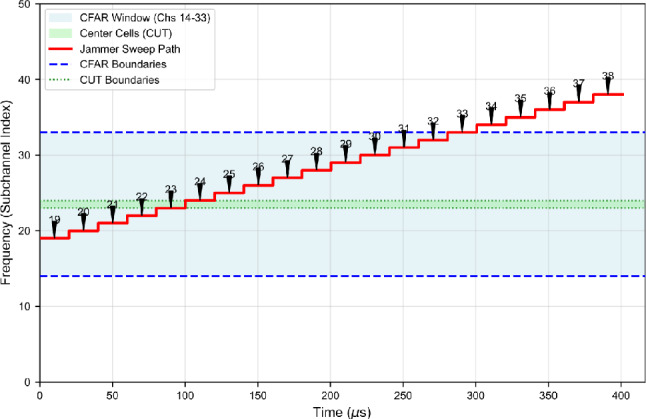
Time-frequency representation of wide-sweep interference affecting 20 of 50 subchannels.

**Fig. 10:**
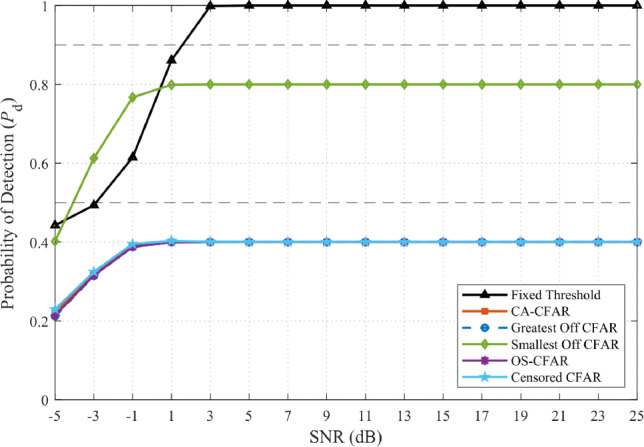
CFAR detection and false-alarm performance under wide-sweep interference (JSR = 1 dB, NU = 1 dB).

### The security paradox: when robustness becomes vulnerability

The exceptional resilience of frequency-domain CFAR detectors against diverse interference sources introduces a fundamental cybersecurity paradox. The same adaptive thresholding that guarantees reliable detection under hostile jamming conditions simultaneously resists intentional access-denial or administrative control signals. In essence, the mechanism designed to safeguard spectrum sensing becomes a shield against legitimate spectrum management actions.

Simulation results demonstrate that even under severe wide-sweep interference–contaminating up to 40% of the monitored band–CFAR-equipped secondary users (SUs) maintain nonzero detection probability across all tested SNR levels. This persistence arises because the CFAR mechanism continuously re-estimates local noise power and scales its detection threshold accordingly:

49 $$\begin{aligned} \lambda _{c_0} = \alpha Z_{c_0}, \qquad \mathbb {P}(T_{c_0}> \lambda _{c_0} | \mathcal {H}_0) = P_{\textrm{FA}}. \end{aligned}$$

As a result, increasing interference power merely raises both $Z_{c_0}$ and $\lambda _{c_0}$ proportionally, preserving the Neyman–Pearson false-alarm constraint and leaving the detector’s operational capability intact. Conventional jamming–whether broadband, swept-FM, or noise-based–fails to suppress such adaptive sensing without simultaneously degrading legitimate PU reception.

This dual-use behavior has serious security implications. A CFAR-equipped adversary can exploit its adaptivity to maintain covert or unauthorized links, effectively neutralizing network-level denial mechanisms. In military, emergency, or restricted-access communication networks, this property could allow untrusted SUs to persist within contested spectral environments despite administrative attempts to suspend their activity. The conventional approach of jamming to minimize $P_d$ is fundamentally flawed: it risks creating missed detections that lead to harmful interference with licensed PU transmissions–precisely the outcome spectrum management seeks to prevent.

Therefore, the very robustness that defines CFAR’s advantage in hostile or uncertain environments transforms into a potential vulnerability in security-sensitive systems. Adaptive detectors that ”ride the interference floor” are inherently resistant not only to accidental interference but also to deliberate control signals. This paradox necessitates a paradigm shift from traditional jamming strategies to *intelligent counter-CFAR mechanisms*–approaches that exploit the detector’s own statistical dependencies to enforce administrative control. Rather than attempting to hide PU signals (low $P_d$), the solution lies in creating deceptive occupancy conditions that maximize false alarms ($P_{fa}$) while maintaining high detection of genuine PUs ($P_d$). This strategy, detailed in the following section, leverages algorithm-aware interference shaping to selectively neutralize adaptive SUs while preserving legitimate PU operations and spectrum integrity.

## Administrator-controlled counter-access via comb-sweep jamming

The established resilience of frequency-domain CFAR detectors, while beneficial for legitimate SUs, introduces the security paradox central to this work: this same adaptivity can be exploited by malicious users to evade administrative control. The conventional jamming forms reviewed in Section "Background and related work"—barrage, spot, and sweep-spot interference—each attempt to defeat the receiver through raw power; none can systematically suppress an adaptive detector that rescales its threshold in real time. A fundamentally different counter-access strategy is therefore required. Rather than attacking the physical-layer reception, the proposed approach targets possibly malicious or illegitimate SUs by exploiting the CFAR detector’s own statistical dependencies to manipulate its perception of the spectral environment.

### Strategic framework: deceptive occupancy

The proposed counter-access framework is engineered to create a persistent illusion of spectrum occupancy, a strategy we term *deceptive occupancy*. Unlike traditional jamming, which aims to reduce the probability of detection ($P_d$) and risks causing harmful interference with legitimate primary transmissions, our approach pursues a dual objective that leverages the asymmetric costs inherent in cognitive radio networks.

The first objective is to maximize the probability of false alarm ($P_{fa}$), forcing the SU to perceive vacant channels as occupied and thus denying opportunistic access. Concurrently, the second objective is to maintain a high probability of detection ($P_d$), ensuring that genuine PU signals remain detectable to prevent harmful interference. This strategy recognizes that the cost of missed detection–which risks interference with critical licensed services–far exceeds the cost of false alarms, which merely denies spectrum access to opportunistic users.

The mechanism achieves these two objectives by strategically contaminating the CFAR detector’s reference windows with structured interference. When jammer energy falls within the reference cells $\mathcal {R}(c_0)$, it inflates the noise estimate $Z_c$ computed by the SU’s detector. Since the adaptive threshold is set as $\lambda _c = \alpha Z_c$, where $\alpha$ is the scaling constant for the target false alarm rate, the contaminated estimate yields an elevated threshold that causes the detector to misclassify noise as signal (high $P_{fa}$), while the PU’s signal power in the CUT remains sufficiently dominant to exceed $\lambda _c$ (maintaining high $P_d$).

This approach effectively decouples the conventional $P_d$–$P_{fa}$ trade-off inherent in Neyman-Pearson detection theory. Rather than operating along a fixed receiver operating characteristic (ROC) curve, the administrator manipulates the SU’s environmental conditions–shifting the detector to a high-$P_{fa}$ regime through reference window contamination while the true system $P_d$ remains robust due to the preserved dominance of PU signal power.

### Analytical signal model and design optimization

The administrator-controlled jammer generates two simultaneous narrowband tones that sweep periodically across the unallocated spectrum. The time-domain signal model for $N_t = 2$ tones is given by:

50 $$\begin{aligned} j(t) = \sqrt{\frac{2P_J}{N_t}} \sum _{i=1}^{2} \cos (2\pi [f_c + (i-1)\Delta f + f_{\text {sweep}}(t)]t + \phi _i), \end{aligned}$$

where $P_J$ is the total jamming power distributed across both tones, $f_c$ is the starting frequency of the first tone, $\Delta f = 25B_{ch}$ is the tone spacing set to 25 times the subchannel bandwidth for enhanced spectral coverage, $f_{\text {sweep}}(t)$ defines the time-varying frequency offset over the sweep period, and $\phi _i$ are independent random phases ensuring noncoherent addition of the tones.

The instantaneous spectrum of the comb-sweep interference is expressed as:

51 $$\begin{aligned} J(f,t) = \sum _{i=1}^{2} \sqrt{\frac{P_J}{2N_t}} \delta \left( f - [f_c + (i-1)\Delta f + f_{\text {sweep}}(t)]\right) , \end{aligned}$$

where $\delta (\cdot )$ is the Dirac delta function. At any instant *t*, two discrete spectral lines occupy specific subbands whose center frequencies evolve according to $f_{\text {sweep}}(t)$, traversing the total monitored bandwidth $B_T$ to ensure periodic contamination of CFAR reference regions.

**Fig. 11:**
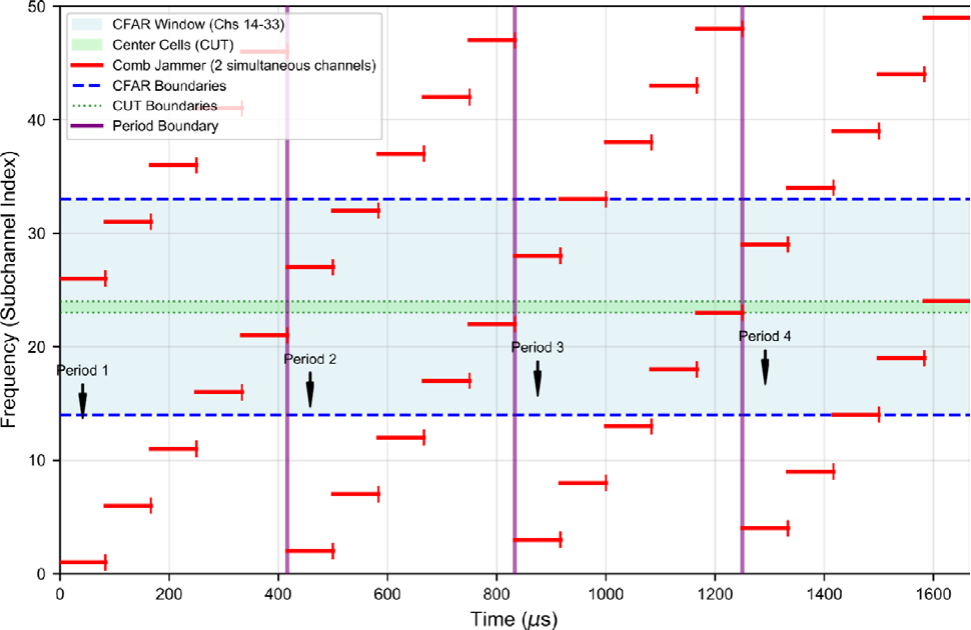
Time-frequency representation of dual-tone comb-sweep jammer over four periods (1600 $\mu$s). The periodic sweep ensures systematic contamination of CFAR reference windows while preserving spectral gaps for PU transmissions.

The jammer operates in a deterministic four-period cycle with total duration $T_{cycle} \approx 1.6$ ms, where each period spans $T_{period} \approx 416$ $\mu$s. The sweep function is:

52 $$\begin{aligned} f_{\text {sweep}}(t) = f_0 + \frac{\Delta f_{sweep}}{T_{period}} \left( t \bmod T_{period}\right) , \end{aligned}$$

where $f_0$ is the initial frequency offset and $\Delta f_{sweep}$ is the total frequency excursion per period. Figure 11 illustrates this sweep pattern within the CFAR sensing window, and Table 5 summarizes the key parameters.

**Table 5:** Comb-Sweep Jammer Configuration Parameters.

Parameter	Value
Number of Simultaneous Tones ($N_t$)	2
Target Subchannel Range	0–50
Total Cycle Duration ($T_{cycle}$)	$\approx$ 1.6 ms
Number of Sweep Periods	4
Period Duration ($T_{period}$)	416 $\mu$s
CFAR Analysis Window	Channels 14–33
Subchannel Bandwidth ($B_{ch}$)	12.5 kHz

The dual-tone configuration with rapid sweep dynamics is engineered to defeat the outlier-rejecting CFAR variants under consideration through algorithm-aware contamination. When a jammer tone occupies a reference cell at index $j \in \mathcal {R}(c_0)$, the cell power is:

53 $$\begin{aligned} \bar{P}_j = \bar{P}_j^{(\text {noise})} + \bar{P}_j^{(\text {jam})}, \end{aligned}$$

where $\bar{P}_j^{(\text {noise})}$ is the background noise power and $\bar{P}_j^{(\text {jam})}$ is the jammer contribution. For mean-level estimators such as CA-CFAR, this directly inflates the noise estimate:

54 $$\begin{aligned} Z_{CA} = \frac{1}{|\mathcal {R}(c_0)|} \sum _{j \in \mathcal {R}(c_0)} \bar{P}_j, \end{aligned}$$

where $|\mathcal {R}(c_0)| = 2N_r$ is the total number of reference cells.

Order-statistic detectors like OS-CFAR are designed to resist contamination by rejecting outliers. The OS-CFAR variant uses the *r*-th order statistic with $r=12$ from $|\mathcal {R}(c_0)|=16$ reference cells, discarding the four strongest signals:

55 $$\begin{aligned} Z_{OS} = \bar{P}_{(r=12)}. \end{aligned}$$

The jammer exploits this parameterization through temporal dynamics. By sweeping its two tones with period $T_{period} \approx 416$ $\mu$s, it contaminates up to four distinct reference cells within a single SU observation window. In realistic conditions with two active PU signals, the total number of strong signals becomes six (four from jammer, two from PUs), exceeding OS-CFAR’s rejection capacity. After discarding the four strongest signals, $Z_{OS}$ remains based on contaminated cells containing jammer or PU energy rather than true noise. Similarly, Censored-CFAR ($M_c = 4$) is overwhelmed when censoring removes four powers but six strong signals are present.

The contaminated noise estimate inflates the adaptive threshold:

56 $$\begin{aligned} \lambda _{c_0} = \alpha Z_{c_0}, \end{aligned}$$

ensuring persistent elevated thresholds. The rapid sweep through all unallocated channels prevents any frequency from remaining interference-free long enough for reliable SU spectrum sensing, creating the perception of continuous occupancy.

### Performance evaluation of controlled interference

The effectiveness of the comb-sweep jamming strategy depends critically on selecting the optimal JSR that balances two competing objectives: maximizing false alarm probability to deny SU spectrum access while maintaining high detection probability to protect primary users. Unlike conventional CFAR design, which accepts an inherent trade-off between $P_{fa}$ and $P_d$ through threshold adjustment, this work exploits environmental manipulation to decouple these metrics. However, determining the optimal JSR requires systematic evaluation across the feasible operating space, as excessive jamming power risks threshold over-inflation that degrades PU detectability, while insufficient power permits intermittent SU access.

Systematic optimization requires exploring the two-dimensional parameter space $(JSR, \alpha )$ to identify the global optimum that balances competing enforcement objectives. The JSR determines the physical interference power affecting both detection and false alarm metrics across all CFAR variants, while $\alpha$ represents the administrator’s strategic weighting between PU protection (detection) and SU denial (false alarm induction). Neither parameter can be optimized independently—the effectiveness of a given JSR depends critically on the chosen weighting strategy, and conversely, the optimal weighting depends on the available jamming power.

To quantify this trade-off rigorously across the joint parameter space, a general performance metric $\zeta (JSR, \alpha )$ is formulated that combines detection and false alarm characteristics. For each variant *i* and performance metric $P_x \in \{P_d, P_{fa}\}$, a consistency-weighted measure is computed as:

57 $$\begin{aligned} M_{P_x,i} = \text {median}(P_{x,i}) \times (1 - \text {IQR}(P_{x,i})) \end{aligned}$$

where $P_{x,i}$ represents the vector of probabilities evaluated across the SNR range of $-5$ to 25 dB. The median captures central tendency while the $(1 - \text {IQR})$ term penalizes variability, where $\text {IQR} = Q_3 - Q_1$ denotes the interquartile range. Since $P_x$ values lie in [0, 1], the IQR naturally bounds to [0, 1], making $(1-\text {IQR})$ a consistency penalty that approaches zero for highly variable performance and unity for perfectly consistent performance. This formulation is preferred over simple averaging because the median provides robustness against outliers while the IQR penalty ensures that performance consistency across the SNR range is valued equally with high absolute performance.

The optimization objective is then defined as:

58 $$\begin{aligned} \zeta (\text {JSR}, \alpha ) = \alpha \ln \left( \sum _{i=1}^{N} M_{P_d,i}\right) + (1-\alpha ) \ln \left( \sum _{i=1}^{N} M_{P_{fa},i}\right) \end{aligned}$$

where *N* denotes the number of CFAR variants under evaluation. The logarithmic transformation ensures balanced sensitivity to both metrics by preventing either term from dominating through magnitude alone, effectively penalizing configurations where either detection or denial fails catastrophically. A comprehensive grid search evaluates $\zeta (\text {JSR}, \alpha )$ over the full two-dimensional space: JSR $\in [-10, -1]$ dB in 1 dB increments and $\alpha \in [0.1, 0.9]$ in 0.1 increments, yielding 90 distinct operating points. Each configuration is evaluated via Monte Carlo simulation with 10000 iterations under the system assumptions detailed in Section "Analytical signal model and design optimization". Note that $\alpha$ represents the administrator’s weighting strategy in the optimization framework, not an SU-controlled parameter; secondary receivers employ fixed CFAR scaling factors designed for nominal $P_{fa} = 0.1$ under interference-free conditions.

**Fig. 12:**
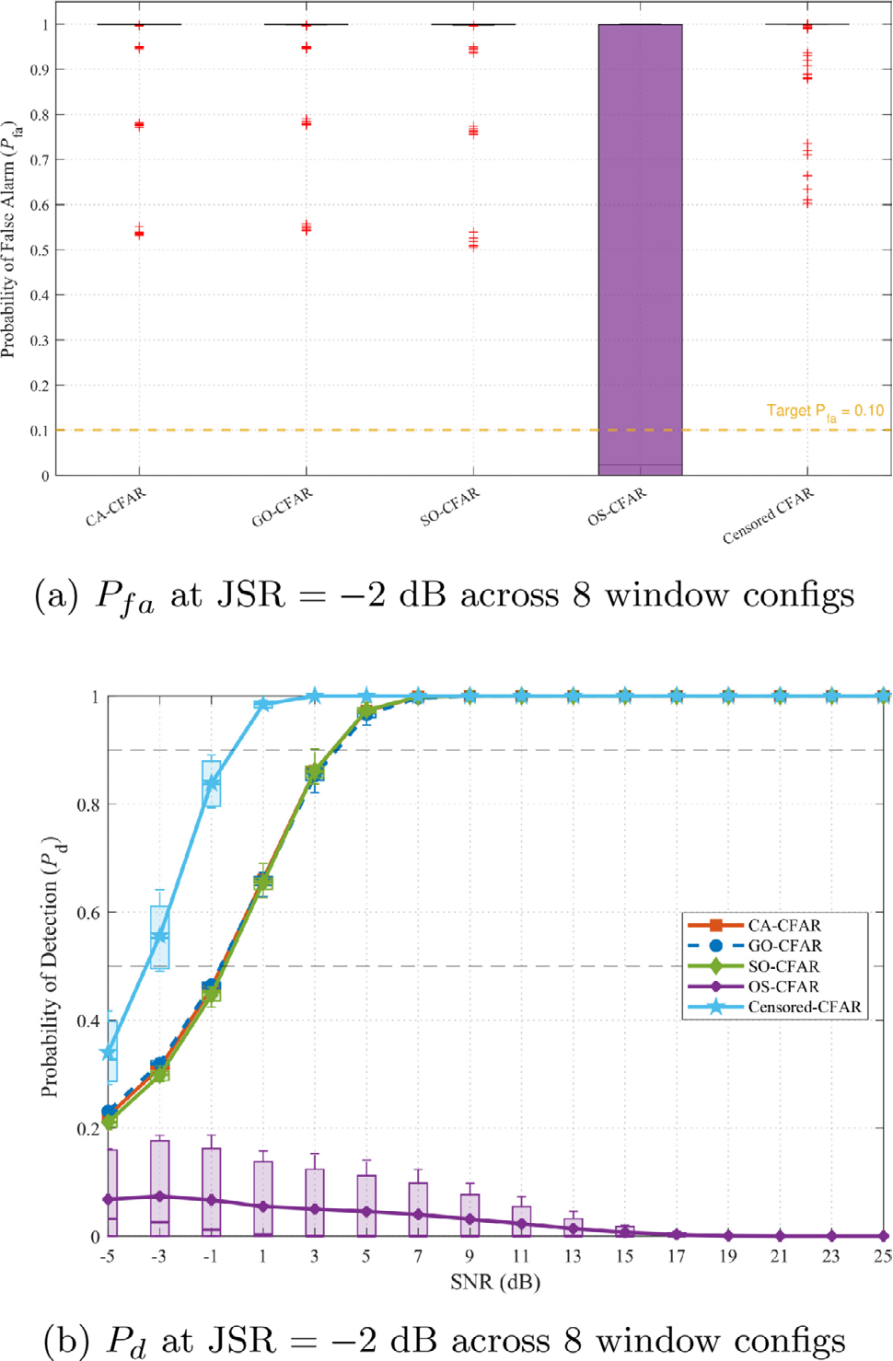
Performance at optimal JSR $= -2$ dB across eight CFAR window configurations (Table 6). Each box spans the distribution over all configurations and SNR range $[-5, 25]$ dB. Narrow IQR across configurations confirms geometry-independence of the enforcement mechanism.

**Fig. 13:**
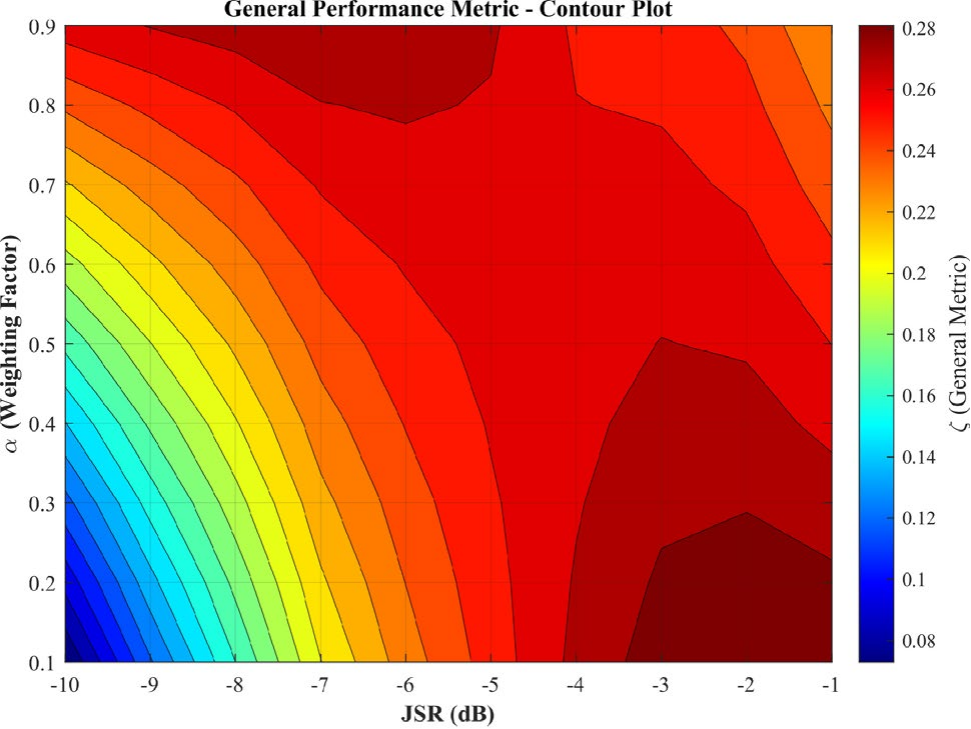
Optimization landscape with OS-CFAR included, showing bifurcated structure with primary optimum at JSR $= -2$ dB, $\alpha = 0.1$ and secondary region at JSR $\in [-9, -5]$ dB, $\alpha \in [0.8, 0.9]$.

Figure 13 presents the optimization landscape when all five CFAR variants (including OS-CFAR) are evaluated. The contour reveals a complex, bifurcated structure with two distinct operating regions. The primary region at high JSR ($-3$ to $-1$ dB) and low $\alpha$ (0.1 to 0.2) exhibits the global maximum at (JSR $= -2$ dB, $\alpha = 0.1$), as confirmed by Fig. 12. To assess robustness under two practically important uncertainty scenarios—first, an adversary that deliberately randomises its CFAR window parameters to evade enforcement, and second, an administrator who lacks prior knowledge of the illegal SU’s sensing configuration—these figures display performance aggregated across eight practical window configurations (reference cells per side $N_r \in \{6, 8, 10, 12, 14, 16, 18, 20\}$, guard cells $N_g \in \{0, 1, 2\}$; see Table 6). Each box in the plot spans the distribution of the metric across all eight configurations and the full SNR range ($-5$ to 25 dB). A secondary region emerges at lower JSR ($-9$ to $-5$ dB) with elevated $\alpha$ (0.8 to 0.9), prioritizing detection uniformity over complete denial. This bifurcation arises from OS-CFAR’s fundamentally different response to jamming: at high JSR, its detection collapses catastrophically while false alarm reaches unity; at moderate JSR, detection remains functional but denial is incomplete ($P_{fa} \approx 0.26$). The optimization metric $\zeta$ thus identifies two qualitatively different enforcement philosophies, neither dominating across all operational contexts.

**Table 6:** CFAR window configurations evaluated in sensitivity analysis.

Config.	$N_r$/side	$N_g$/side	Total window
1	6	0	13
2	8	0	17
3	10	1	23
4	12	1	27
5	14	1	31
6	16	2	37
7	18	2	41
8	20	2	45

This complex landscape motivates re-evaluation of OS-CFAR’s inclusion in the threat model. For unauthorized secondary users seeking reliable spectrum access under administrator-controlled jamming, OS-CFAR presents fundamental operational liabilities. At the administrator’s global optimum (JSR $= -2$ dB), OS-CFAR experiences detection collapse with $P_d$ approaching 0.25 at operational SNR, rendering it ineffective for locating PU-vacant spectrum despite achieving $P_{fa} = 1.0$. At moderate JSR values where OS-CFAR maintains detection capability, its $P_{fa} \approx 0.26$ provides only partial access—inferior to mean-level CFARs’ performance. Critically, an unauthorized user cannot predict the administrator’s JSR selection, creating strategic uncertainty: OS-CFAR’s utility depends entirely on environmental parameters outside the SU’s control. A rational SU requires both high $P_d$ (to locate opportunities) and low $P_{fa}$ (to avoid wasted transmission), yet OS-CFAR cannot guarantee both metrics simultaneously under comb-sweep jamming. This JSR-dependent unpredictability, combined with catastrophic failure modes at the administrator’s preferred operating point, renders OS-CFAR unsuitable as a robust sensing algorithm for unauthorized spectrum access in contested environments where algorithm-aware countermeasures are deployed.

**Fig. 14:**
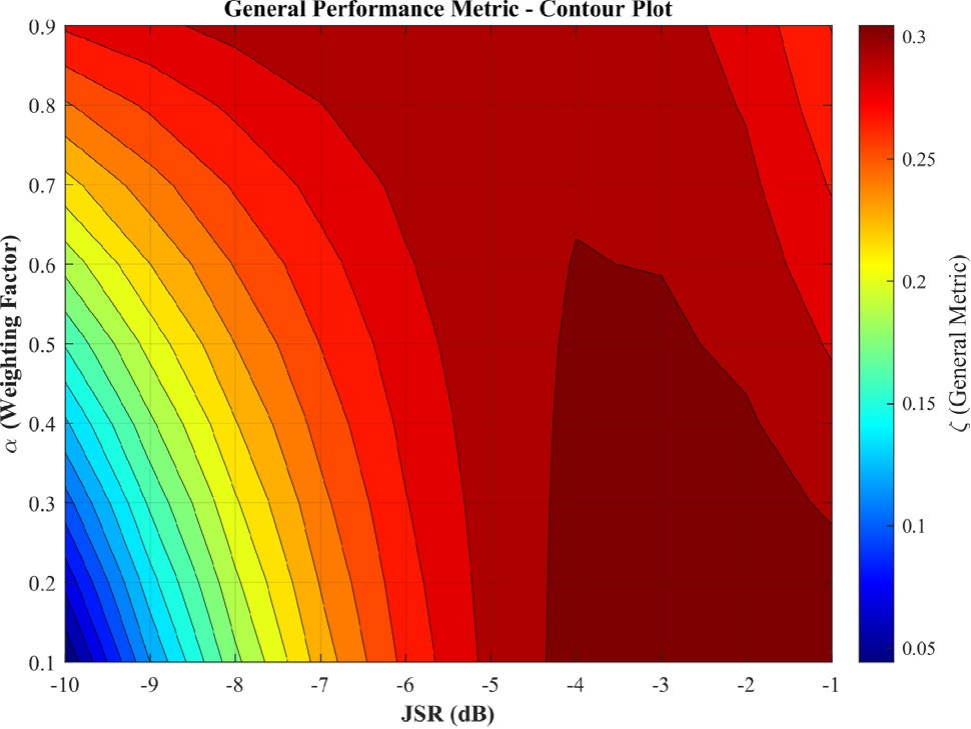
Optimization landscape excluding OS-CFAR, revealing unified optimal region at JSR $\in [-5, -1]$ dB with consistent performance plateau.

From the administrator’s perspective, excluding OS-CFAR from the optimization simplifies enforcement strategy without sacrificing effectiveness against realistic threats. Figure 14 presents the optimization landscape when only the five mean-level variants (CA-CFAR, GO-CFAR, SO-CFAR, Censored-CFAR, Fixed Threshold) are evaluated. The bifurcated structure collapses into a single, unified optimal region spanning JSR $\in [-5, -1]$ dB with consistent peak performance across $\alpha \in [0.1, 0.6]$. This consolidation offers three critical operational advantages. First, the elimination of competing optima removes strategic ambiguity—the administrator faces a clear design space rather than a choice between qualitatively different enforcement philosophies. Second, the wide JSR plateau provides robustness against channel uncertainty: performance remains nearly invariant across a 4 dB span from JSR $= -5$ dB to JSR $= -1$ dB, tolerating propagation variability without performance degradation. This tolerance is essential because administrators cannot guarantee precise JSR at the SU receiver due to path loss uncertainty, fading, and unknown receiver characteristics. Third, all five mean-level variants exhibit predictable, homogeneous failure modes under strong jamming: systematic threshold inflation drives $P_{fa} \rightarrow 1.0$ across the entire optimal JSR range, ensuring comprehensive denial regardless of which specific mean-based algorithm the SU employs.

**Fig. 15:**
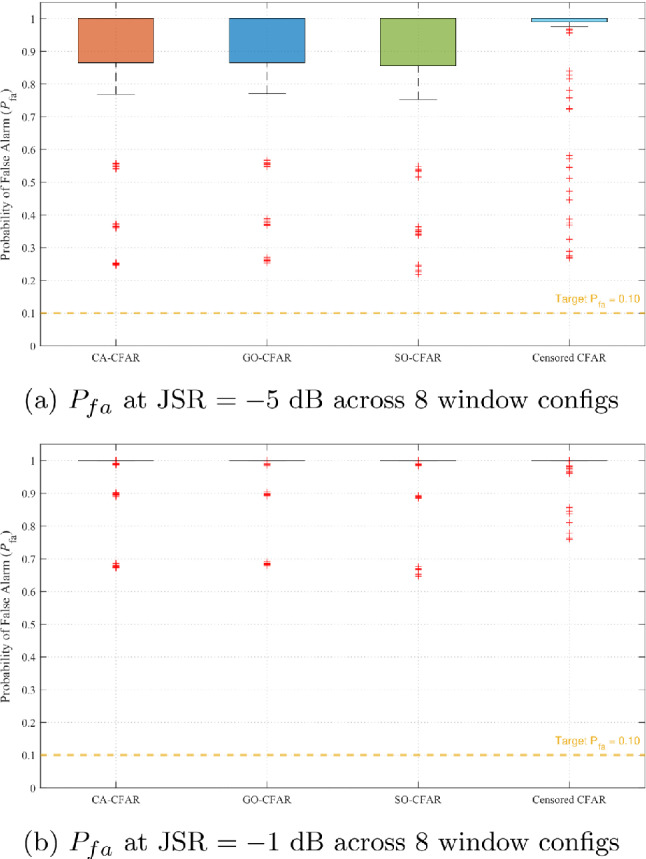
False alarm comparison at optimal region boundaries (OS-CFAR excluded). Each box spans all eight CFAR window configurations (Table 6) and the full SNR range. Narrow IQR confirm that neither JSR variation nor window-parameter randomisation provides any evasion benefit.

**Fig. 16:**
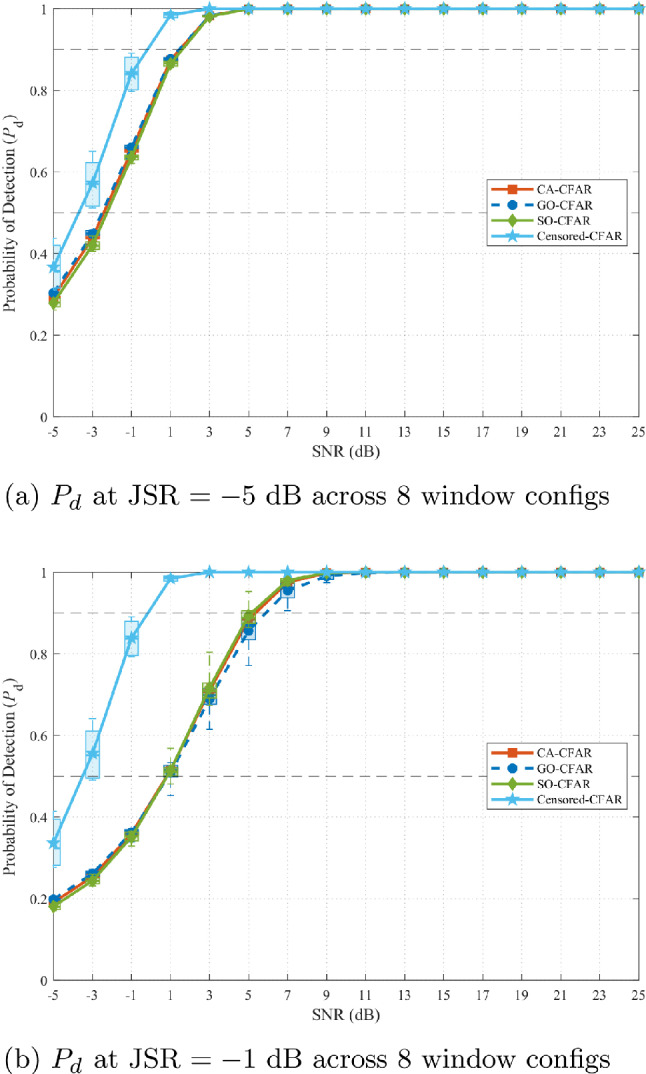
Detection comparison at optimal region boundaries (OS-CFAR excluded). Each box spans all eight CFAR window configurations (Table 6) and the full SNR range. Narrow IQR confirms that window-parameter randomisation provides no detection advantage to the adversary, and PU protection is preserved throughout.

To validate both the JSR robustness claim and the geometry-independence of the enforcement mechanism, Figs. 15 and 16 compare performance at the extremes of the optimal region: JSR $= -5$ dB and JSR $= -1$ dB (OS-CFAR excluded). In each figure, each box spans the distribution of the corresponding metric across all eight CFAR window configurations listed in Table 6 and the full SNR range $[-5, 25]$ dB, so that the width of the box directly quantifies how much a malicious SU could gain by randomising its window parameters. Figure 15 demonstrates that false alarm induction remains uniformly effective across both JSR values *and* across all window configurations. At JSR $= -5$ dB (Fig. 15a), all five mean-level variants achieve median $P_{fa} = 1.0$ with IQR [0.97, 1.0] regardless of window geometry, indicating that no configuration offers an evasion advantage. At JSR $= -1$ dB (Fig. 15b), IQR narrows further to [1.0, 1.0] across all variants and all configurations. This double invariance—across JSR within the plateau and across window geometry—confirms that the comb-sweep mechanism’s effectiveness depends solely on the power balance $P_{\textrm{PU}}/P_{\textrm{jam}}$, not on the adversary’s algorithmic choices.

Figure 16 reveals that detection performance maintains consistency across both the JSR range and all window configurations while preserving PU protection. At JSR $= -5$ dB (Fig. 16a), Censored-CFAR achieves $P_d \approx 1.0$ at SNR $= 1$ dB, and CA-CFAR, GO-CFAR, and SO-CFAR reach $P_d \approx 0.97$ at the same SNR, with unity attained by SNR $= 3$ dB. Critically, the IQR across all eight window configurations is bounded within $\pm 0.02$ probability for every variant, confirming that the SU gains no detection improvement by switching window parameters. At JSR $= -1$ dB (Fig. 16b), the detection distributions overlay within the same $\pm 0.02$ bound. All variants maintain $P_d \ge 0.65$ at SNR $= 1$ dB and reach unity by SNR $= 6$ dB or earlier, ensuring reliable PU protection throughout the operational SNR range regardless of window geometry.

The validation demonstrates that the optimal region functions as a true performance plateau, providing a dual form of robustness: invariance across the 4 dB JSR span *and* invariance across the full practical range of CFAR window geometries. An administrator can select any JSR value within $[-5, -1]$ dB and any SU can randomise $N_r \in \{6,\ldots ,20\}$ or $N_g \in \{0,1,2\}$—in neither case does the enforcement outcome change. Complete SU denial ($P_{fa} = 1.0$) is achieved with maintained PU protection ($P_d \ge 0.65$ at SNR $= 3$ dB). The 4 dB JSR tolerance accommodates real-world propagation uncertainties including path loss variability ($\pm 2$ dB typical for land mobile radio), shadowing effects, and unknown SU receiver implementation losses. The $\pm 0.02$ geometry tolerance confirms that enforcement effectiveness is insensitive both to which specific mean-level CFAR algorithm the SU implements and to the window parameters it selects.

### Integration with P25 protocol timing

The 1.6 ms cycle duration is engineered to align with APCO Project 25 (P25) control channel timing structures^42^. This synchronization serves a dual purpose. First, it prevents the SU from identifying predictable interference-free windows that could be exploited for opportunistic sensing. Second, the rapid cycle makes the jammer activity indistinguishable from legitimate fast-paced state changes in digital trunked radio systems^43^.

A sophisticated adversary might attempt to learn the jammer’s pattern and conduct spectrum sensing during gaps. However, by matching the 416 $\mu$s period to typical P25 slot durations^42^, the administrator ensures each SU sensing interval encounters interference, precluding the construction of a reliable spectral occupancy model. This asymmetric information advantage–the administrator possesses detailed knowledge of network timing while the SU does not–transforms the jamming signal from a detectable anomaly into an apparent component of normal network operation^44^.

The comb-sweep strategy thus represents a practical implementation of policy-driven spectrum enforcement. By exploiting CFAR’s adaptive mechanism and leveraging protocol-layer timing, the administrator regains control over spectrum access without compromising PU communications or violating emission constraints.

To quantify the cost of this enforcement, we analyse spectrum availability at the optimal operating point (JSR $= -2$ dB). The dual-tone sweep must contaminate approximately 75–80% of the monitored band to guarantee systematic inflation of every CFAR reference window. This leaves **20–25%** of the 50 subchannels–roughly 10–13 slots–available for legitimate PU transmissions, while from the SU’s perspective all subchannels appear occupied ($P_{fa} = 1.0$), yielding **0% apparent availability**. This 20–25% ceiling is not an arbitrary design choice but a consequence of a fundamental *power-balance constraint*: to simultaneously achieve $P_{fa}\rightarrow 1.0$ (SU denial) and maintain high $P_d$ (PU protection), the jammer must occupy the majority of CFAR reference cells. Increasing PU density beyond this bound reduces the relative jammer contribution to reference windows, causing $P_{fa}$ to fall below the denial threshold; compensating by raising $P_{\textrm{jam}}$ risks threshold over-inflation that collapses $P_d$ for legitimate PUs. This inherent trade-off between enforcement strength and spectrum utilisation is an important operational limitation that administrators must account for when deploying the comb-sweep countermeasure.

## Conclusion and future work

This paper presented a dual investigation into robust spectrum sensing and administrative counter-access in cognitive radio networks. We confirmed that advanced CFAR detectors, particularly mean-level variants such as SO-CFAR and Censored-CFAR, offer significant resilience against conventional jamming, yet this robustness enables malicious users to exploit them for unauthorized spectrum access. To address this vulnerability, we introduced an algorithm-aware countermeasure: a comb-sweep jammer that effectively denies spectrum access to all mean-level CFARs (CA, GO, SO, Censored) by systematically contaminating their reference windows, thereby driving $P_{fa} \rightarrow 1.0$ while maintaining PU protection ($P_d$) across a robust 4 dB JSR plateau ($-5$ to $-1$ dB). Quantitative analysis reveals that this enforcement retains approximately 20–25% of subchannels for legitimate PU use, while achieving complete SU denial, an outcome that remains unchanged across all tested window sizes spanning reference cell counts from 6 to 20 and guard cell counts of 0 to 2, leaving no exploitable parameter degree of freedom for the adversary. Conversely, OS-CFAR’s outlier-rejection mechanism exhibits complex, JSR-dependent trade-offs that render it unreliable for attackers, validating an enforcement strategy focused on the predictable failure of mean-level CFARs. Future work will investigate hybrid CFAR architectures integrating different estimation logics to maintain high-integrity detection under intensified jamming, with FPGA/SDR validation for deployment in contested electromagnetic environments.

## Data Availability

All data generated or analysed during this study are included in this published article. The findings are based entirely on Monte Carlo simulations, with all parameters and configurations fully described within the manuscript, making the results independently reproducible.
